# Role of Cell Division Autoantigen 1 (CDA1) in Cell Proliferation, Fibrosis

**DOI:** 10.3390/genes1030335

**Published:** 2010-09-30

**Authors:** Ban-Hock Toh, Yugang Tu, Zemin Cao, Mark E. Cooper, Zhonglin Chai

**Affiliations:** 1 Autoimmunity Laboratory, Centre for Inflammatory Diseases, Department of Medicine, Faculty of Medicine, Nursing, Health Sciences, Monash University, Clayton, Victoria 3168, Australia; 2 Diabetes, Metabolism Division, Baker IDI Heart, Diabetes Institute, 75 Commercial Rd, Melbourne, Victoria 3004, Australia; E-Mails: Yugang.Tu@bakeridi.edu.au (Y.T.); Zemin.Cao@bakeridi.edu.au (Z.C.); Mark.Cooper@bakeridi.edu.au (M.E.C.); Zhonglin.Chai@bakeridi.edu.au (Z.C.)

**Keywords:** TGF-β, tumorigenesis, DNA damage, atherosclerosis, diabetes mellitus

## Abstract

Cell Division Autoantigen 1 (CDA1) was discovered following screening a human expression library with serum from a patient with Discoid Lupus Erythematosus. CDA1, encoded by TSPYL2 on the X chromosome, shares anti-proliferative, pro‑fibrotic properties with TGF-β. It inhibits cell growth through p53, pERK1/2, p21‑mediated pathways, is implicated in tumorigenesis, the DNA damage response. Its pro-fibrotic property is mediated through cross-talk with TGF-β that results in upregulation of extracellular matrix proteins. The latter properties have identified a key role for CDA1 in diabetes associated atherosclerosis. These dual properties place CDA1 as an attractive molecular target for treating tumors, vascular fibrosis including atherosclerosis, other vascular disorders associated with enhanced TGF-β action, tissue scarring.

## 1. Introduction

Anti-nuclear autoantibodies (ANA) are useful diagnostic markers of systemic autoimmune diseases [1]. As ANAs typically react with highly conserved epitopes of biologically important nuclear antigens, they have also been useful for the molecular cloning, functional characterization of their cognate nuclear autoantigens. Thus, ANAs played pivotal roles in the molecular characterization of the extractable nuclear antigen “Sm” as components of the splicosome, of proliferating cell nuclear antigen, “PCNA”, as a molecule implicated in cell proliferation. 

Discoid lupus erythematosus (DLE) is primarily a cutaneous subset of systemic lupus erythematosus associated with low titer ANAs in up to 40% of patients [2]. We found that serum from a patient with DLE contains not only autoantibodies to phosphoepitopes of mitotic chromosomes [3] but also to a nuclear phosphoprotein that we named CDA1 (Cell Divison Autoantigen 1) [4].

## 2. Molecular Cloning of CDA1 Using an Autoimmune DLE Serum

We cloned CDA1 by using the DLE serum to screen a human testis cDNA expression library. Its open reading frame comprises a cDNA of 2,079 base pairs encoding 693 amino acids with predicted molecular mass of 79,430 daltons [4]. Localization of CDA1 to the nucleus was based on the following observations [4]: Its predicted amino acid sequence contains four putative nuclear localization signals (NLSs); it localized to the nuclear but not cytoplasmic fraction of HeLa cells by immunoblotting; Myc-tagged or enhanced green fluorescent protein-tagged CDA1;, its N-terminal segment containing its four NLSs localized to the nucleus, nucleolus of transfected HeLa cells. Nuclear localization of CDA1 is supported by the study of Ueki et al. [5], They reported that a partial cDNA clone (GenBankTM accession number AB015345) encoding aa 208–693 of CDA1 that included its third, fourth NLS localized to the nucleus of COS-7 cells. Thus the third, fourth NLS are sufficient to target CDA1 to the nucleus while the role of the first, second remains unknown.

## 3. CDA1 in Man, Mouse

CDA1 is encoded by TSPYL2 (TSPY-like 2, a homolog of TSPY on the X chromosome), a member of the testis-specific protein Y-encoded, TSPY-like/SET/nucleosome assembly protein-1 superfamily [6]. CDA1 has also been described as cutaneous T-cell lymphoma-associated antigen se20-4 [7], DENTT (Differentially Expressed Nucleolar TGF-beta1 Target) [8], NP79 (a 79Kda nuclear protein encoded by a gene expressed in samples of normal hearts, hearts with common congenital defects) [9]. A mouse homologue of CDA1 was named CASK-interacting nucleosome assembly protein (CINAP). It was reported as a co-transcription factor in neurons with CASK binding, nucleosome assembly protein activity [10]. 

## 4. Domain Structure of CDA1

**Figure 1:**
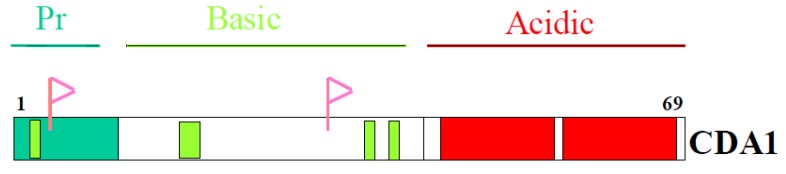
Domain structure of CDA1 showing proline-rich domain (Pr), basic, acidic domains with four nuclear localization signals (bars), two consensus phosphorylation sites (flags).

CDA1 has 693 amino acid residues with three structural domains (Figure 1): An N-terminal proline-rich domain, a central basic domain,, a C-terminal acidic domain. The four NLSs reside in the N-terminal proline-rich domain, central basic domain.

## 5. CDA1 Requires its C-Terminal Domain for Anti-Proliferative Activity

Endogenous CDA1 levels are low in resting serum starved HeLa cells, dramatically elevated in serum stimulated cells. These observations suggest a role for CDA1 in cell growth. We explored its role in cell growth in stable HeLa cell lines transfected with Myc-tagged CDA1 in which CDA1 levels were regulated by a tetracyclin-off promoter [5]. Maximal expression of CDA1 transgene observed after 3–5 days of culture without tetracyclin was followed by dramatic arrest of cell growth, cell density after 4–5 days of culture. DNA synthesis assessed by BrdUrd uptake showed a gradual decline in the first three days followed by a virtual complete arrest by days 4–5. Incremental increases in CDA1 expression regulated by decreasing doxycycline concentrations, was accompanied by a corresponding incremental decrease of HeLa colony numbers, cell growth, cell density,, BrdUrd uptake. These observations indicate the level of CDA1 transgene expression, regulated cell growth, DNA synthesis. The inhibitory effects of CDA1 were not accompanied by a change in cell viability assessed by trypan blue dye exclusion, nor in cell cycle profiles assessed by flow cytometry. The results suggest that CDA1 may exert inhibitory effects at multiple stages of the cell cycle, acting as a negative regulator of cell cycle progression. The suggestion is consistent with the elevated levels of endogenous CDA1 in G1, S,, M phases of the cell cycle. 

The acidic C-terminal tail of CDA1 is required for its inhibitory effects on cell growth, DNA synthesis as stable HeLa cell transfectants lacking this domain did not arrest cell growth or DNA synthesis. Thus transfectants with its N-terminal domain only, comprising of nucleotides 132–1,487, failed to affect cell growth [5]. The acidic C-terminal tail of CDA1 shares ∼40% identity, 68% similarity with the acidic C‑terminal tail of the leukemia associated protein SET [5]. The central region of CDA1 shares the same level of identity, similarity with most of the remainder of SET. SET is a 32-kDa ubiquitous nuclear protein identified fused to CAN in acute undifferentiated leukemia [11]. CAN has also been found fused with DEK in a subtype of acute myeloid leukemia [12], suggesting that CAN is an oncogene activated by fusion with SET or DEK. The only common motif of SET, DEK is their acidic domain. SET is a potent, specific inhibitor of protein phosphatase 2A [13]. 

The action of CDA1 in inhibiting cell growth is supported by observations that CDA1/DENTT inhibited the growth of human, mouse tumors, cell lines [14], that its levels are upregulated in growth-arrested Jurkat T cells [15]. That CDA1 also regulates the growth, development of Toxoplasma gondii [16] suggests its growth inhibitory activity is evolutionarily conserved, a finding consistent with other nuclear autoantigens [1]. The ability of CDA1 to inhibit cell growth also required phosphorylation of its two consensus Cdk phosphorylation sites. A CDA1 double mutant with both phosphorylation sites mutated to alanine (S20A, T340A), disabled its capacity to inhibit cell growth, failed to inhibit DNA synthesis in S‑phase cells [4]. These observations suggest that in addition to its expression levels, phosphorylation of these two sites by cyclin/CDKs play important roles in regulating its role in cell proliferation. Cyclin D/Cdk4, cyclin A/Cdk2,, cyclin B/Cdk1 phosphorylated CDA1 *in vitro* at either or both phosphorylation sites, whereas cyclin E/CDK2 did not. Phospho-amino acid analysis confirmed phosphorylation of both serine, threonine sites by cyclin A/Cdk2 [4]. Our studies suggest that CDA1 may be differentially phosphorylated throughout the cell cycle on serine 20, threonine 340 to regulate cell proliferation. 

## 6. Proline-Rich N Terminal Domain of CDA1

CDA1 contains an N-terminal Pr domain not present in SET or other related proteins. Pr domains bind to Src Homology 3 (SH3) domains of intracellular, membrane-associated proteins, kinases [17,18] involved in signal transduction. The amino acid sequence motif of Pr domains for SH3 domain binding is P*XX*P with neighboring residues forming patterns specific for individual SH3 domain of different proteins [19]. CDA1 has three regions containing P*XX*P motifs, although no specific patterns are found for binding to known SH3 domains. The region also contains stretches of nine, five P residues. CDA1 may interact, through its Pr domain, with unique cognate protein(s), the identification of which may provide more information on its function.

## 7. CDA1 Regulates p21 Expression

Cell cycle progression is controlled by kinase activities of Cdks complexed with specific cyclins at certain cell cycle phases. These activities are negatively regulated by Cdk inhibitor proteins (CdkIs) of the Cip/Kip, INK4 families. The two families of Cdk inhibitors inactivate different classes of Cdks [20,21]. The Cip/Kip family member, p21^Waf1/Cip1^ (p21) was first identified in a quaternary complex containing D cyclin, Cdk,, PCNA [22]. p21 inhibits cell proliferation, activities of several cyclin-Cdk complexes *in vitro* [23]. The tumor suppressor protein p53 controls transcriptional regulation of the p21 gene by binding to the p53 responsive distal region of the p21 promoter in response to intracellular signals such as DNA damage [24,25,26]. TGF-β also transcriptionally regulates p21 by binding to the TGF-β responsive element at the proximal region, which does not necessarily require functional p53 in HaCat cells [27]. The MEK/ERK intracellular signaling pathway upregulates p21 in response to stimulation by several factors including TGF-β [28]. 

Using the Tet-Off HeLa cell line where CDA1 levels can be regulated by doxycycline concentrations [4] we have provided the following compelling data implicating CDA1 in regulating p21 transcription, expression [29]: A progressive time-, dose-dependent increase in CDA1 protein, mRNA levels was accompanied by a corresponding but delayed progressive increase in mRNA, protein expression of p21; a progressive increase followed by a subsequent decrease in CDA1 levels, was also matched by a corresponding rise, fall in p21 levels; CDA1 overexpression activated the p21 promoter in a luciferase reporter assay.

Inhibiting CDA1 transgene overexpression after 48 h gradually decreased CDA1 protein levels, indicating a relatively long half-life of CDA1 protein in these cells. When CDA1 protein, p21 mRNA levels dropped to a level similar to that seen endogenously, cell growth arrest was released, allowing cells to proliferate again [29]. This indicates that CDA1-overexpressing cells remained viable, consistent with our earlier report [4]. More importantly, this finding demonstrated that cell growth arrest, p21 induction are both quantitatively dependent on CDA1 expression.

## 8. CDA1 is an Upstream Regulator of p53 Expression

p53 is induced by DNA damage that results in transcription of p21. We found that p53 is also induced by CDA1 overexpression, an effect lost with CDA1 knockdown by siRNA [29]. The observation that CDA1 inactivates the ubiquitin ligase for p53 degradation (MDM2), without affecting p53 mRNA levels, suggests that the increased p53 levels are due to increased protein stability. MDM2’s ability to degrade p53 is positively regulated by phosphorylation at S166 by Akt [30]. As CDA1 also inactivates Akt, it suggests a mechanism contributing to MDM2 inactivation [29]. Structural studies of the p21 gene promoter revealed a major p53 responsive element located <2.4 kb upstream of the p21 gene transcription start site [31]. This has been confirmed by global screening of p53 binding sites in the human genome [32]. The p21 promoter with deletion of the distal p53 responsive element (p21(p53-)-Luc) largely lost its basal, almost 100% of its responsive activities to CDA1 overexpression in HeLa cells. P53 knockdown by siRNA attenuated p21 induction by CDA1. The data suggest CDA1 induces p21 gene transcription by p53 binding to its distal responsive element of the p21 gene promoter. 

These observations suggest that CDA1 may have a key role as an upstream regulator of p53 in the DNA damage response where p53 mediates cell cycle arrest, or apoptosis [33,34,35,36,37,38,39]. To test this hypothesis we treated cells with CPT, a molecule that inhibits DNA topoisomerase I by trapping it in cleavable complexes within transcribed chromosomal regions leading to irreversible DNA breaks [40,41]. CPT treatment indeed increased CDA1 mRNA, protein expression levels, induced p53 protein induction while CDA1 knockdown attenuated CPT-induced p53 induction [29]. This is the first report demonstrating CDA1 as an essential upstream regulator of p53 in the DNA damage response. The exact mechanism whereby CDA1 is up-regulated by DNA damage remains unclear.

## 9. CDA1 Activates ERK1/MAPK Pathways

Mild stimulation of the residual activity of p21(p53-)-Luc by CDA1 overexpression raised the possibility of other responsive elements in the downstream region of the p53 responsive element. MEK/ERK1/2 MAPK pathway may contribute to this residual activity as inhibition of this pathway by two MEK inhibitors, PD98059, U0126, blocked the transcriptional induction of p21, p21 promoter activity stimulated by CDA1 [29]. These data are consistent with reports of induction of p21 gene expression by this signaling pathway [42,43,44,45]. Thus, it is likely that both p53, activated ERK1/2 MAPK activate the p21 gene by binding to its promoter. However the target of activated ERK1/2 MAPK on the p21 gene promoter remains unknown. ERK1/2 phosphorylation was induced by CDA1 overexpression suggesting that this may be responsible for activating the ERK1/2 MAPK pathway. A gradual decrease in ERK1/2 phosphorylation while CDA1 is still expressed at a high level probably reflects negative regulation of ERK1/2 by dual-specificity MAP kinase phosphatases [46,47]. Given the nuclear localization of CDA1, its overexpression may modulate upstream kinases such as MEK, Raf, Ras,, cell surface receptor kinases linked to the MEK/1/2 MAPK pathway. However, it is possible that CDA1 can shuttle between the cytoplasm, nucleus since CDA1, also known as DENTT, has been detected in the cytoplasm, nucleus or both in certain tissues of adult mouse, monkey [48,49]. 

**Figure 2:**
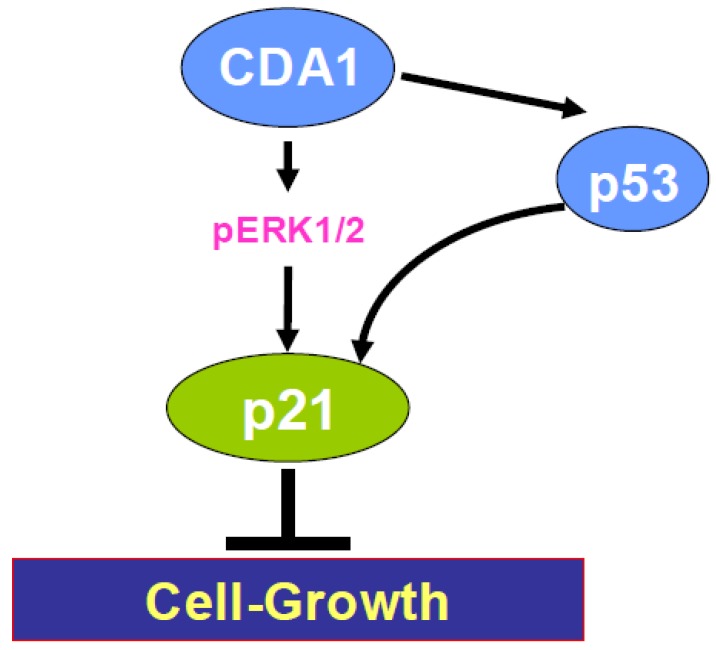
CDA1 inhibits cell growth through upregulation of p21 mediated by p53, pERK1/2. These activities of CDA1 appear to be mediated by binding of p53, pERK1/2 to binding sites on the p21 gene promoter as outlined in Figure 3.

Thus, CDA1 appears to upregulate p21 expression through upregulation of p53, phosphorylation of ERK1/2, as schematized in Figure 2.

**Figure 3:**
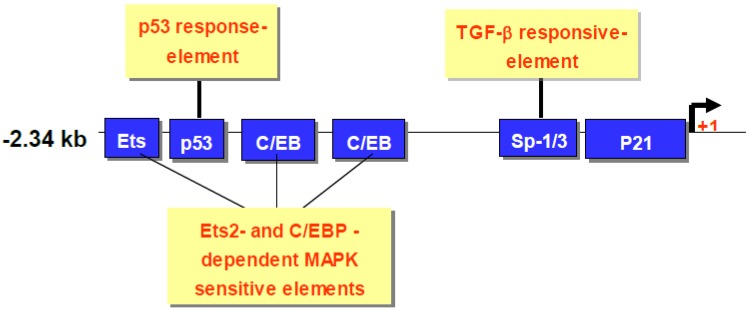
Schematic representation of p21 binding sites for p53, pERK1/2, TGFβ responsive elements.

## 10. CDA1 Inhibits Cyclin-Dependent Kinases Through Upregulation of CDK Inhibitors

p21 expression induced by CDA1 is accompanied by inhibition of the activity of Cdk2, Cdk1 activity, consistent with its binding to cyclins, Cdks,, the cyclin-Cdk complex [50,51,52,53,54]. The data are consistent with our report that CDA1 overexpression inhibits DNA synthesis [4], an activity dependent on S-phase Cdks. However, CDA1 overexpression also inhibits the activities of Cdk4, Cdk6. These activities cannot be attributed solely to induction of p21 expression as p21 plays a positive role in regulating activities of the D type cyclins‑Cdk 4/6 [55,56,57,58], by promoting formation of active cyclin/Cdk complexes. It seems likely therefore that CDA1 overexpression may have also induced other Cdk inhibitor proteins that, together with p21, are potent inhibitors of G1, G1/S, S,, G2/M-phase Cdks. 

## 11. CDA1 is Upregulated in Diabetes-Associated Atherosclerosis

Diabetes mellitus aggravates atherosclerosis-based cardiovascular disease [59]. Excess accumulation of extracellular matrix (ECM) leading to vascular fibrosis may contribute to increased plaque size, leading to luminal narrowing [60,61,62,63]. 

Streptozotocin induced diabetic *ApoE*^−/−^ mouse is a well characterized animal model of diabetes accelerated atherosclerosis. These mice develop increased vascular ECM accumulation associated with rapid development of atherosclerotic plaques similar to human atherosclerosis [62,63,64,65,66]. 

Using this model, we found that after 10 weeks of diabetes mRNA levels of the CDA1-encoding gene *Tspyl2* and profibrotic growth factors *Tgf-β* and *Ctgf* increased 1.5 to 2-fold in aortas of *ApoE*^−/−^ mice. [67]. At 20 weeks of diabetes, expression of these genes increased by more than 5-fold (*Tspyl2*), 10-fold (*Tgf-β*), 15-fold (*Ctgf*), compared with age matched non-diabetic animals. These findings are consistent with our previous reports [62,63]. Our observation suggests that CDA1 is induced at an earlier stage, before the disease becomes manifest, consistent with a role for CDA1 in the development, progression of diabetes associated atherosclerosis in these mice. 

TGF-β is the major growth factor implicated in vascular matrix accumulation via the SMAD signaling pathway [27,68,69,70]. TGF-β, together with angiotensin II, AGE, glucose, mechanical stretch are enhanced in the diabetic vasculature. These agents likely stimulate ECM accumulation, vascular remodeling through TGF-β-dependent, -independent signaling pathways. Targeting downstream signaling of TGF-β could therefore be beneficial in reducing ECM accumulation. Indeed, blockade of the renin-angiotensin system with drugs such as ACE inhibitors reduced fibrosis, attenuated diabetes associated atherosclerosis, partly via TGF-β-dependent pathways [62]. However, targeting TGF-β mediated ECM accumulation in the atherosclerotic plaque at a later stage remains controversial, as it may decrease the fibrous cap, increase the risk of plaque rupture [28,71]. 

## 12. CDA1, TGF Cross-Talk

The MAPK pathway, transcriptional upregulation of p21^Waf1/Cip1^ are both regulated by TGF‑β [28,71,72,73]. Cross-talk between CDA1, TGF-β is supported by the observation that CDA1 is upregulated by TGF-β, that CDA1 increased luciferase activities of TGF-β reporter constructs in some lung cancer cell lines [8,48,49]. These findings implicate CDA1 in TGF-β signaling, its biological functions. The pro-fibrotic activity of TGF-β has been suggested to play a central role in the promotion of diabetic complications, such as nephropathy, macrovascular diseases [72,74].

We carried out studies in vascular smooth muscle cells (VSMCs) to address the hypothesis that an increase in CDA1-encoding gene *Tspyl2* expression in diabetic atherosclerosis enhances the level of TGF-β that promotes ECM accumulation, TGF-β treatment rapidly increased CDA1 protein levels followed by SMAD3 phosphorylation. CDA1 protein levels gradually decreased for 1 to 2 h after peaking, rising again at 4 to 5 h. The first increase in CDA1 is apparently a result of exogenous TGF-β stimulation. The second peak in CDA1 at 4 to 5 h may reflect an autocrine effect of endogenous TGF-β, consistent with a previous observation that the protein level of CDA1 is increased by TGF-β treatment at 4, 8 h in a human lung cell line 

Overproduction of the CDA1-encoding gene *Tspyl2* in VSMCs increased expression of *Ctgf* and ECM genes, including collagen I, III, IV,, fibronectin. Conversely, siRNA-mediated knockdown of *Tspyl2* markedly abrogated TGF-β-dependent expression of *Ctgf* and ECM genes. Our data clearly show that CDA1 promotes the pro-fibrotic effects of TGF-β in the development of atherosclerosis in diabetes. 

TGF-β mediates its effect on vascular fibrosis [75], diabetic nephropathy [76,77] through SMAD3. Knockdown of *Tspyl2*, the gene encoding CDA1, also attenuated TGF-β-stimulated SMAD3 phosphorylation, the stimulatory effect of TGF-β on its target genes associated with ECM production, including *Ctgf*, collagen I, III, IV,, fibronectin. The observations support the potential efficacy of targeting CDA1 to inhibit or reduce ECM production arising from enhanced TGF-β action coordinated by elevated CDA1 production. Targeting TGF-β or TGF-β receptors result in deleterious effects due to inhibition of its other biological effects. In contrast, targeting CDA1 would most likely block only the pro-fibrotic action of TGF-β.

**Figure 4:**
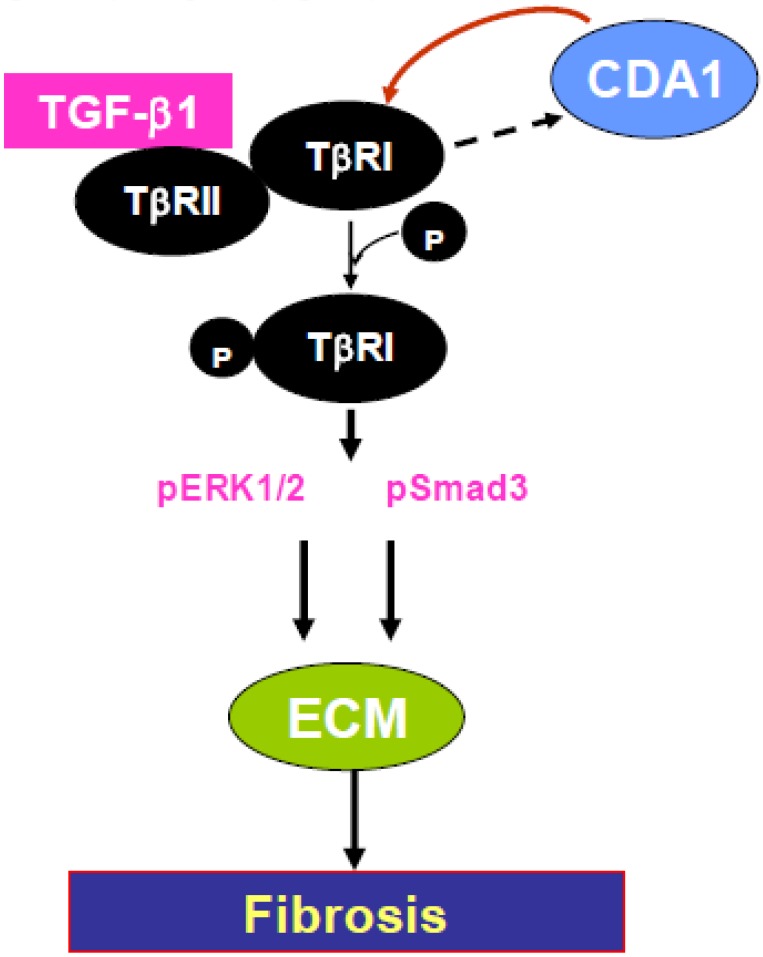
Schematic representation of the promotion of fibrosis arising from cross-talk between CDA1, TGF-β

TGF-β receptor type I has dual specificity kinase activities that phosphorylate R-SMAD proteins including SMAD3 [78,79], activate the ERK MAPK pathway. This is consistent with a role for CDA1, not only in enhancing TGF-β-stimulated SMAD3 activity, but also in activating ERK MAPK, a, pathway implicated in diabetic complications [80]. As crosstalk between SMAD, MAPK pathways plays an important role in fibrosis in the diabetic vasculature, kidney [81], we suggest that the cross-talk is mediated by CDA1 acting through TGF-β receptor 1 (Figure 4).

## 13. Conclusions

CDA1 shares anti-proliferative, pro-fibrotic properties with TGF-β. Our findings suggest that CDA1 plays a key role in these TGFβ-dependent activities. These properties of CDA1 implicate CDA1 as a negative regulator of tumorigenesis,, as a key player in p53-dependent DNA damage responses, fibrotic diseases. Targeting CDA1 may therefore be effective in the control of tumor growth, in attenuating the profibrotic action of TGF-β. Therefore, CDA1 appears to be an attractive molecular target for treating neoplasia, vascular fibrosis including atherosclerosis, other vascular disorders associated with enhanced TGF-β action, tissue scarring as commonly seen in diabetes.
